# Absolute configuration of odorine

**DOI:** 10.1107/S1600536810034227

**Published:** 2010-08-28

**Authors:** Hoong-Kun Fun, Suchada Chantrapromma, Orapun Yodsaoue, Chatchanok Karalai

**Affiliations:** a X-ray Crystallography Unit, School of Physics, Universiti Sains Malaysia, 11800 USM, Penang, Malaysia; b Crystal Materials Research Unit, Department of Chemistry, Faculty of Science, Prince of Songkla University, Hat-Yai, Songkhla 90112, Thailand; c Department of Chemistry, Faculty of Science, Prince of Songkla University, Hat-Yai, Songkhla 90112, Thailand

## Abstract

The title compound, known as odorine or roxburghiline {systematic name: (*S*)-*N*-[(*R*)-1-cinnamoylpyrrolidin-2-yl]-2-methyl­butanamide}, C_18_H_24_N_2_O_2_, is a nitro­genous compound isolated from the leaves of *Aglaia odorata*. The absolute configuration was determined by refinement of the Flack parameter with data collected using Cu *K*α radiation showing positions 2 and 2′ to be *S* and *R*, respectively. The pyrrolidine ring adopts an envelope conformation. In the crystal, mol­ecules are linked into chains along [010] by inter­molecular N—H⋯O hydrogen bonds.

## Related literature

For ring conformations, see: Cremer & Pople (1975 ▶). For standard bond-length data, see: Allen *et al.* (1987 ▶). For background to the *Aglaia* plants and their biological activity, see: Brader *et al.* (1998 ▶); Cui *et al.* (1997 ▶); Engelmeier *et al.* (2000 ▶); Hayashi *et al.* (1982 ▶); Inada *et al.* (2001 ▶); Nugroho *et al.* (1999 ▶); Purushothaman *et al.* 1979 ▶); Saifah *et al.* (1993 ▶); Shiengthong *et al.* (1979 ▶). For related structures, see: Babidge *et al.* (1980 ▶); Dumontet *et al.* (1996 ▶); Hayashi *et al.* (1982 ▶). For the stability of the temperature controller used in the data collection, see Cosier & Glazer (1986 ▶).
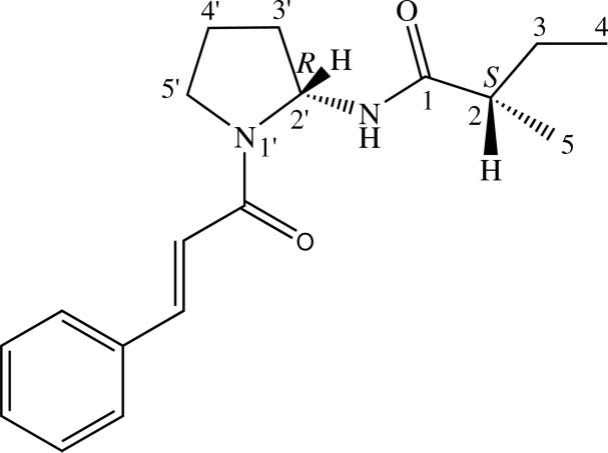


## Experimental

### 

#### Crystal data

C_18_H_24_N_2_O_2_
*M* *_r_* = 300.39
Monoclinic, 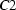

*a* = 18.8909 (3) Å
*b* = 6.8398 (1) Å
*c* = 13.4174 (2) Å
β = 107.054 (1)°
*V* = 1657.43 (4) Å^3^
*Z* = 4
Cu *K*α radiation
μ = 0.63 mm^−1^
*T* = 100 K
0.57 × 0.16 × 0.13 mm

#### Data collection

Bruker APEXII DUO CCD area-detector diffractometer
Absorption correction: multi-scan (*SADABS*; Bruker, 2009 ▶) *T*_min_ = 0.718, *T*_max_ = 0.924
10656 measured reflections
2625 independent reflections
2606 reflections with *I* > 2σ(*I*)
*R*_int_ = 0.042

#### Refinement

*R*[*F* ^2^ > 2σ(*F* ^2^)] = 0.033
*wR*(*F* ^2^) = 0.096
*S* = 1.16
2625 reflections
205 parameters
1 restraint
H atoms treated by a mixture of independent and constrained refinement
Δρ_max_ = 0.21 e Å^−3^
Δρ_min_ = −0.27 e Å^−3^
Absolute structure: Flack (1983 ▶), 1036 Friedel pairs
Flack parameter: 0.03 (18)

### 

Data collection: *APEX2* (Bruker, 2009 ▶); cell refinement: *SAINT* (Bruker, 2009 ▶); data reduction: *SAINT*; program(s) used to solve structure: *SHELXTL* (Sheldrick, 2008 ▶); program(s) used to refine structure: *SHELXTL*; molecular graphics: *SHELXTL*; software used to prepare material for publication: *SHELXTL* and *PLATON* (Spek, 2009 ▶).

## Supplementary Material

Crystal structure: contains datablocks global, I. DOI: 10.1107/S1600536810034227/lh5122sup1.cif

Structure factors: contains datablocks I. DOI: 10.1107/S1600536810034227/lh5122Isup2.hkl

Additional supplementary materials:  crystallographic information; 3D view; checkCIF report

## Figures and Tables

**Table 1:** Hydrogen-bond geometry (Å, °)

*D*—H⋯*A*	*D*—H	H⋯*A*	*D*⋯*A*	*D*—H⋯*A*
N2—H1*N*2⋯O1^i^	0.81 (2)	2.09 (2)	2.8789 (16)	163 (2)

Symmetry code: (i) 
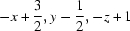
.

**Fig. 1.**
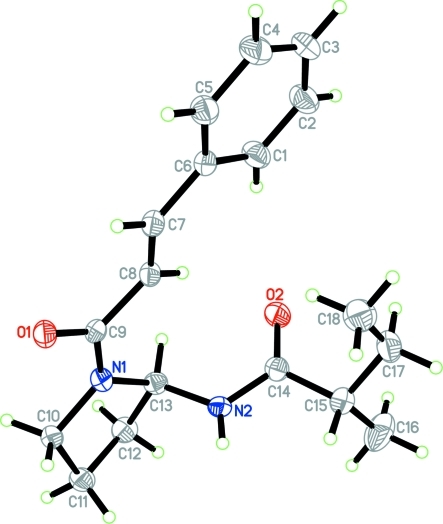
The molecular structure of (I), showing 50% probability displacement ellipsoids and the atom-numbering scheme.

**Fig. 2.**
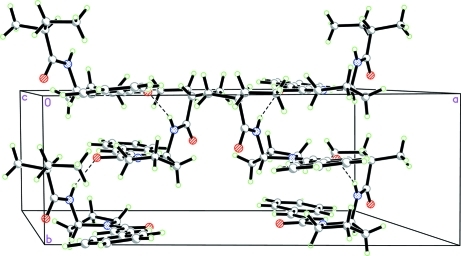
The crystal packing of (I) viewed approximately along the c axis, showing one dimensional chains along [0 1 0]. N—H···O hydrogen bonds are shown as dashed lines.

## supplementary crystallographic information

### Comment

Several species of the genus *Aglaia* of the family Meliaceae are of ethnomedicinal values and have insecticidal (Brader *et al.*, 1998), antifungal (Engelmeier *et al.*, 2000) and cytotoxic (Saifah *et al.*, 1993; Cui *et al.*, 1997) activities. These interesting activities have prompted us to screen for further bioactive compounds from *Aglaia odorata*. *Aglaia odorata* known locally in Thai as "Pra-yong" or "Hom-glai" is a small tree occurring primarily in South-East Asia. The leaves and roots of this plant have been used in local folk medicine as a heart stimulant, febrifuge and to retrieve toxin by causing vomiting. The isolated compounds from this plant also show interesting biological activities such as anticancer (Inada *et al.*, 2001), insecticidal (Nugroho *et al.*, 1999) and anti-leukemic (Hayashi *et al.*, 1982) activities. In the course of our research of chemical constituents and bioactive compounds from the leaves of *A. odorata* which were collected from Songkhla province in the southern part of Thailand, the title aminopyrrolidine odorine (I), also known as odorine (Shiengthong *et al.*, 1979) or roxburghiline or *N*-cinnamoyl-2-(2-methylbutanoylamino)pyrrolidine (Purushothaman *et al.*, 1979) was isolated. The previous report showed that (I) possesses cancer-chemopreventive activity (Inada *et al.*, 2001). The absolute configuration of (I) was determined by making use of the anomalous scattering of Cu Kα*X*-radiation with the Flack parameter being refined to 0.03 (18). We report herein the crystal structure of (I).

Fig. 1 shows that the molecule of (I) possesses a 2-aminopyrrolidine ring linked by two amide functions to 2-methylbutyric acid and cinnamic acid. The pyrrolidine ring adopts an envelope conformation with the puckered C12 atom having a deviation of 0.223 (1) Å and with the puckering parameters Q =  0.3522 (15) Å and θ = 281.3 (2)° (Cremer & Pople, 1975). Atoms of the cinnamoyl (C1–C9/O1) moiety essentially lie on the same plane (*r.m.s*. 0.0216 (1) Å) with a max. deviation of 0.0583 (1) Å for atom O1. Atoms C13, C14, C15, N2 and O2 lie on the same plane (*r.m.s*. 0.0216 (1) Å). The mean plane through C13/C14/C15/N2/O2 makes the dihedral angle of 88.80 (7)° with the mean plane through the cinnamoyl moiety. The 2-methylbutanamide chain at C13 is pseudo-axial with the C14–N2–C13–C12 torsion angle  = 125.55 (13)°. The orientation of the butyl group is described by the torsion angle C14–C15–C17–C18 = 68.78 (18)°. The bond distances in (I) are within normal ranges (Allen *et al.*, 1987) and comparable with the related structures which are odorinol (Hayashi *et al.*, 1982) and forbaglin A (Dumontet *et al.*, 1996). The absolute configuration at atoms C15 and C13 or positions 2 and 2' of the odorine are *S*,*R* configurations which agree with the previous stereochemistry of odorine (Babidge *et al.*, 1980).

In the crystal packing of (I) (Fig. 2), the molecules are linked into chains along [010] through N2—H1N2···O1^i^ hydrogen bonds (Fig. 2 and Table 1).

### Experimental

Ground-dried leaves of *A. odorata* (53.70 g) were extracted with CH_2_Cl_2_ and CH_3_OH (each of 2 *x* 2 *L*) for a duration of 3 days at room temperature. The solvents were evaporated under reduced pressure to afford CH_2_Cl_2_ (23.50 g) and CH_3_OH (5.23 g) crude extracts, respectively. The CH_2_Cl_2_ crude extract (23.50 g) was further purified by quick column chromatography (QCC) over silica gel using hexane as eluent and increasing polarity with EtOAc and CH_3_OH to afford 10 fractions (F1-F10). Fraction F10 (231.6 mg) was further separated by column chromatography with acetone-hexane (3:7), yielding the title compound as white solid (15.6 mg). Colorless needle-shaped single crystals of the title compound suitable for *x*-ray structure determination were recrystallized from CH_2_Cl_2_ by the slow evaporation of the solvent at room temperature after several days, Mp. 476-478 K.

### Refinement

The amide H atom was located in a difference map and refined isotropically. The remaining H atoms were placed in calculated positions with (C—H) = 0.98 for CH, 0.97 for CH_2_ and 0.96 Å for CH_3_ atoms. The *U*_iso_ values were constrained to be 1.5*U*_eq_ of the carrier atom for methyl H atoms and 1.2*U*_eq_ for the remaining H atoms. A rotating group model was used for the methyl groups. The highest residual electron density peak is located at 0.73 Å from C6 and the deepest hole is located at 0.71 Å from C9. 1036 Friedel pairs were used to determine the absolute configuration.

### Crystal data

**Table:**

C_18_H_24_N_2_O_2_	*F*(000) = 648
*M**_r_* = 300.39	*D*_x_ = 1.204 Mg m^−^^3^
Monoclinic, *C*2	Melting point = 476–478 K
Hall symbol: C 2y	Cu *K*α radiation, λ = 1.54178 Å
*a* = 18.8909 (3) Å	Cell parameters from 2625 reflections
*b* = 6.8398 (1) Å	θ = 6.8–67.4°
*c* = 13.4174 (2) Å	µ = 0.63 mm^−^^1^
β = 107.054 (1)°	*T* = 100 K
*V* = 1657.43 (4) Å^3^	Needle, colorless
*Z* = 4	0.57 × 0.16 × 0.13 mm

### Data collection

**Table:**

Bruker APEXII DUO CCD area-detector diffractometer	2625 independent reflections
Radiation source: sealed tube	2606 reflections with *I* > 2σ(*I*)
graphite	*R*_int_ = 0.042
φ and ω scans	θ_max_ = 67.4°, θ_min_ = 6.8°
Absorption correction: multi-scan (*SADABS*; Bruker, 2009)	*h* = −22→22
*T*_min_ = 0.718, *T*_max_ = 0.924	*k* = −7→6
10656 measured reflections	*l* = −16→16

### Refinement

**Table:**

Refinement on *F*^2^	Secondary atom site location: difference Fourier map
Least-squares matrix: full	Hydrogen site location: inferred from neighbouring sites
*R*[*F*^2^ > 2σ(*F*^2^)] = 0.033	H atoms treated by a mixture of independent and constrained refinement
*wR*(*F*^2^) = 0.096	*w* = 1/[σ^2^(*F*_o_^2^) + (0.0595*P*)^2^ + 0.2381*P*] where *P* = (*F*_o_^2^ + 2*F*_c_^2^)/3
*S* = 1.16	(Δ/σ)_max_ = 0.001
2625 reflections	Δρ_max_ = 0.21 e Å^−^^3^
205 parameters	Δρ_min_ = −0.27 e Å^−^^3^
1 restraint	Absolute structure: Flack (1983), 1036 Friedel pairs
Primary atom site location: structure-invariant direct methods	Flack parameter: 0.03 (18)

### Special details

**Table:**

Experimental. The crystal was placed in the cold stream of an Oxford Cryosystems Cobra open-flow nitrogen cryostat (Cosier & Glazer, 1986) operating at 100.0 (1) K.
Geometry. All esds (except the esd in the dihedral angle between two l.s. planes) are estimated using the full covariance matrix. The cell esds are taken into account individually in the estimation of esds in distances, angles and torsion angles; correlations between esds in cell parameters are only used when they are defined by crystal symmetry. An approximate (isotropic) treatment of cell esds is used for estimating esds involving l.s. planes.
Refinement. Refinement of F^2^ against ALL reflections. The weighted R-factor wR and goodness of fit S are based on F^2^, conventional R-factors R are based on F, with F set to zero for negative F^2^. The threshold expression of F^2^ > 2sigma(F^2^) is used only for calculating R-factors(gt) etc. and is not relevant to the choice of reflections for refinement. R-factors based on F^2^ are statistically about twice as large as those based on F, and R- factors based on ALL data will be even larger.

### Fractional atomic coordinates and isotropic or equivalent isotropic displacement parameters (Å^2^)

**Table:**

	*x*	*y*	*z*	*U*_iso_*/*U*_eq_	
O1	0.68617 (5)	0.99471 (16)	0.55658 (7)	0.0243 (2)	
O2	0.97777 (6)	0.88734 (17)	0.73395 (8)	0.0315 (3)	
N1	0.79446 (6)	0.99195 (19)	0.51776 (8)	0.0218 (2)	
H1N2	0.8859 (10)	0.691 (3)	0.5433 (14)	0.030 (5)*	
N2	0.90244 (6)	0.78710 (19)	0.57796 (9)	0.0217 (3)	
C1	0.87323 (8)	0.9894 (3)	0.92951 (11)	0.0299 (3)	
H1A	0.9038	1.0059	0.8870	0.036*	
C2	0.90374 (8)	0.9818 (3)	1.03621 (11)	0.0348 (4)	
H2A	0.9548	0.9923	1.0651	0.042*	
C3	0.85882 (8)	0.9585 (3)	1.10083 (11)	0.0325 (4)	
H3A	0.8797	0.9538	1.1728	0.039*	
C4	0.78306 (9)	0.9425 (3)	1.05783 (11)	0.0337 (4)	
H4A	0.7528	0.9273	1.1008	0.040*	
C5	0.75221 (8)	0.9490 (2)	0.95025 (11)	0.0285 (3)	
H5A	0.7012	0.9376	0.9217	0.034*	
C6	0.79653 (7)	0.9725 (2)	0.88465 (10)	0.0233 (3)	
C7	0.76210 (7)	0.9768 (2)	0.77145 (10)	0.0236 (3)	
H7A	0.7107	0.9679	0.7481	0.028*	
C8	0.79691 (7)	0.9920 (2)	0.69898 (9)	0.0219 (3)	
H8A	0.8483	1.0019	0.7190	0.026*	
C9	0.75502 (7)	0.9936 (2)	0.58677 (9)	0.0207 (3)	
C10	0.75678 (7)	0.9890 (2)	0.40514 (9)	0.0233 (3)	
H10A	0.7308	1.1109	0.3826	0.028*	
H10B	0.7217	0.8819	0.3871	0.028*	
C11	0.81922 (8)	0.9613 (2)	0.35579 (10)	0.0275 (3)	
H11A	0.8097	1.0348	0.2914	0.033*	
H11B	0.8253	0.8243	0.3414	0.033*	
C12	0.88773 (8)	1.0396 (2)	0.43814 (11)	0.0276 (3)	
H12A	0.9325	0.9808	0.4303	0.033*	
H12B	0.8912	1.1805	0.4329	0.033*	
C13	0.87590 (7)	0.9813 (2)	0.54206 (10)	0.0222 (3)	
H13A	0.8990	1.0781	0.5956	0.027*	
C14	0.95055 (7)	0.7544 (2)	0.67329 (11)	0.0237 (3)	
C15	0.97075 (8)	0.5412 (2)	0.69919 (11)	0.0293 (3)	
H15A	0.9332	0.4576	0.6527	0.035*	
C16	1.04558 (10)	0.5039 (3)	0.67983 (16)	0.0512 (5)	
H16A	1.0413	0.5272	0.6077	0.077*	
H16B	1.0603	0.3708	0.6971	0.077*	
H16C	1.0821	0.5903	0.7225	0.077*	
C17	0.97552 (9)	0.4934 (3)	0.81232 (12)	0.0356 (4)	
H17A	0.9971	0.3644	0.8290	0.043*	
H17B	1.0085	0.5865	0.8576	0.043*	
C18	0.90143 (10)	0.4977 (3)	0.83531 (12)	0.0406 (4)	
H18A	0.9089	0.4729	0.9081	0.061*	
H18B	0.8696	0.3990	0.7948	0.061*	
H18C	0.8790	0.6238	0.8176	0.061*	

### Atomic displacement parameters (Å^2^)

**Table:**

	*U*^11^	*U*^22^	*U*^33^	*U*^12^	*U*^13^	*U*^23^
O1	0.0229 (5)	0.0260 (6)	0.0230 (4)	0.0040 (4)	0.0051 (3)	0.0031 (4)
O2	0.0301 (5)	0.0298 (6)	0.0277 (5)	−0.0005 (4)	−0.0025 (4)	−0.0062 (5)
N1	0.0222 (5)	0.0231 (6)	0.0193 (5)	0.0013 (5)	0.0045 (4)	0.0013 (5)
N2	0.0225 (5)	0.0209 (7)	0.0202 (6)	−0.0006 (5)	0.0039 (4)	−0.0046 (5)
C1	0.0277 (7)	0.0399 (9)	0.0226 (6)	0.0012 (7)	0.0080 (5)	0.0015 (7)
C2	0.0269 (7)	0.0507 (10)	0.0245 (7)	0.0036 (7)	0.0038 (5)	−0.0006 (8)
C3	0.0384 (8)	0.0390 (10)	0.0180 (6)	0.0045 (7)	0.0050 (5)	0.0014 (6)
C4	0.0379 (8)	0.0409 (11)	0.0253 (7)	0.0019 (7)	0.0137 (6)	0.0029 (6)
C5	0.0263 (7)	0.0324 (9)	0.0264 (7)	0.0017 (6)	0.0074 (5)	0.0016 (6)
C6	0.0268 (6)	0.0209 (8)	0.0217 (6)	0.0049 (6)	0.0063 (5)	0.0022 (6)
C7	0.0234 (6)	0.0214 (7)	0.0246 (7)	0.0038 (6)	0.0048 (5)	0.0019 (6)
C8	0.0228 (6)	0.0194 (7)	0.0215 (6)	0.0031 (6)	0.0032 (5)	0.0014 (6)
C9	0.0239 (6)	0.0153 (7)	0.0221 (6)	0.0031 (6)	0.0053 (5)	0.0014 (6)
C10	0.0275 (6)	0.0218 (7)	0.0187 (6)	0.0032 (6)	0.0037 (5)	0.0011 (6)
C11	0.0354 (7)	0.0280 (8)	0.0201 (6)	0.0028 (6)	0.0098 (5)	0.0017 (6)
C12	0.0304 (7)	0.0277 (8)	0.0276 (7)	−0.0002 (6)	0.0132 (5)	0.0028 (6)
C13	0.0219 (6)	0.0214 (7)	0.0234 (6)	−0.0015 (5)	0.0070 (5)	−0.0018 (6)
C14	0.0174 (6)	0.0291 (9)	0.0244 (7)	0.0013 (5)	0.0058 (5)	−0.0013 (6)
C15	0.0278 (7)	0.0293 (9)	0.0290 (7)	0.0059 (6)	0.0058 (6)	−0.0003 (6)
C16	0.0451 (9)	0.0479 (13)	0.0675 (12)	0.0202 (9)	0.0274 (9)	0.0085 (10)
C17	0.0397 (8)	0.0319 (9)	0.0304 (7)	0.0055 (7)	0.0027 (6)	0.0066 (7)
C18	0.0535 (9)	0.0358 (10)	0.0347 (8)	0.0013 (8)	0.0163 (7)	0.0039 (8)

### Geometric parameters (Å, °)

**Table:**

O1—C9	1.2437 (16)	C10—C11	1.5243 (18)
O2—C14	1.2280 (19)	C10—H10A	0.9700
N1—C9	1.3480 (17)	C10—H10B	0.9700
N1—C10	1.4695 (15)	C11—C12	1.531 (2)
N1—C13	1.4780 (16)	C11—H11A	0.9700
N2—C14	1.3529 (17)	C11—H11B	0.9700
N2—C13	1.451 (2)	C12—C13	1.5284 (18)
N2—H1N2	0.81 (2)	C12—H12A	0.9700
C1—C2	1.3787 (19)	C12—H12B	0.9700
C1—C6	1.401 (2)	C13—H13A	0.9800
C1—H1A	0.9300	C14—C15	1.522 (2)
C2—C3	1.389 (2)	C15—C17	1.529 (2)
C2—H2A	0.9300	C15—C16	1.532 (2)
C3—C4	1.382 (2)	C15—H15A	0.9800
C3—H3A	0.9300	C16—H16A	0.9600
C4—C5	1.3898 (19)	C16—H16B	0.9600
C4—H4A	0.9300	C16—H16C	0.9600
C5—C6	1.3907 (19)	C17—C18	1.519 (2)
C5—H5A	0.9300	C17—H17A	0.9700
C6—C7	1.4667 (17)	C17—H17B	0.9700
C7—C8	1.3282 (18)	C18—H18A	0.9600
C7—H7A	0.9300	C18—H18B	0.9600
C8—C9	1.4813 (17)	C18—H18C	0.9600
C8—H8A	0.9300		
			
C9—N1—C10	120.52 (10)	C10—C11—H11B	111.0
C9—N1—C13	126.73 (10)	C12—C11—H11B	111.0
C10—N1—C13	112.65 (10)	H11A—C11—H11B	109.0
C14—N2—C13	122.40 (12)	C13—C12—C11	104.37 (11)
C14—N2—H1N2	116.5 (13)	C13—C12—H12A	110.9
C13—N2—H1N2	120.9 (13)	C11—C12—H12A	110.9
C2—C1—C6	120.50 (13)	C13—C12—H12B	110.9
C2—C1—H1A	119.7	C11—C12—H12B	110.9
C6—C1—H1A	119.7	H12A—C12—H12B	108.9
C1—C2—C3	120.45 (13)	N2—C13—N1	110.75 (11)
C1—C2—H2A	119.8	N2—C13—C12	114.38 (12)
C3—C2—H2A	119.8	N1—C13—C12	101.95 (10)
C4—C3—C2	119.75 (13)	N2—C13—H13A	109.8
C4—C3—H3A	120.1	N1—C13—H13A	109.8
C2—C3—H3A	120.1	C12—C13—H13A	109.8
C3—C4—C5	119.91 (14)	O2—C14—N2	122.61 (14)
C3—C4—H4A	120.0	O2—C14—C15	122.00 (12)
C5—C4—H4A	120.0	N2—C14—C15	115.36 (12)
C4—C5—C6	120.94 (13)	C14—C15—C17	111.68 (13)
C4—C5—H5A	119.5	C14—C15—C16	107.63 (14)
C6—C5—H5A	119.5	C17—C15—C16	110.13 (13)
C5—C6—C1	118.44 (12)	C14—C15—H15A	109.1
C5—C6—C7	119.43 (12)	C17—C15—H15A	109.1
C1—C6—C7	122.13 (12)	C16—C15—H15A	109.1
C8—C7—C6	126.53 (12)	C15—C16—H16A	109.5
C8—C7—H7A	116.7	C15—C16—H16B	109.5
C6—C7—H7A	116.7	H16A—C16—H16B	109.5
C7—C8—C9	120.87 (11)	C15—C16—H16C	109.5
C7—C8—H8A	119.6	H16A—C16—H16C	109.5
C9—C8—H8A	119.6	H16B—C16—H16C	109.5
O1—C9—N1	120.81 (11)	C18—C17—C15	114.07 (12)
O1—C9—C8	121.80 (11)	C18—C17—H17A	108.7
N1—C9—C8	117.39 (11)	C15—C17—H17A	108.7
N1—C10—C11	104.19 (10)	C18—C17—H17B	108.7
N1—C10—H10A	110.9	C15—C17—H17B	108.7
C11—C10—H10A	110.9	H17A—C17—H17B	107.6
N1—C10—H10B	110.9	C17—C18—H18A	109.5
C11—C10—H10B	110.9	C17—C18—H18B	109.5
H10A—C10—H10B	108.9	H18A—C18—H18B	109.5
C10—C11—C12	103.95 (11)	C17—C18—H18C	109.5
C10—C11—H11A	111.0	H18A—C18—H18C	109.5
C12—C11—H11A	111.0	H18B—C18—H18C	109.5
			
C6—C1—C2—C3	−0.4 (3)	N1—C10—C11—C12	−24.24 (15)
C1—C2—C3—C4	0.2 (3)	C10—C11—C12—C13	35.81 (15)
C2—C3—C4—C5	0.2 (3)	C14—N2—C13—N1	−119.95 (13)
C3—C4—C5—C6	−0.3 (3)	C14—N2—C13—C12	125.55 (13)
C4—C5—C6—C1	0.0 (2)	C9—N1—C13—N2	72.51 (17)
C4—C5—C6—C7	179.40 (15)	C10—N1—C13—N2	−103.96 (13)
C2—C1—C6—C5	0.4 (3)	C9—N1—C13—C12	−165.39 (14)
C2—C1—C6—C7	−179.02 (17)	C10—N1—C13—C12	18.14 (16)
C5—C6—C7—C8	−177.81 (15)	C11—C12—C13—N2	86.96 (14)
C1—C6—C7—C8	1.6 (3)	C11—C12—C13—N1	−32.61 (14)
C6—C7—C8—C9	179.71 (14)	C13—N2—C14—O2	−3.6 (2)
C10—N1—C9—O1	−0.7 (2)	C13—N2—C14—C15	178.31 (11)
C13—N1—C9—O1	−176.95 (14)	O2—C14—C15—C17	41.89 (18)
C10—N1—C9—C8	178.64 (13)	N2—C14—C15—C17	−139.99 (13)
C13—N1—C9—C8	2.4 (2)	O2—C14—C15—C16	−79.11 (18)
C7—C8—C9—O1	5.1 (2)	N2—C14—C15—C16	99.01 (15)
C7—C8—C9—N1	−174.23 (14)	C14—C15—C17—C18	68.78 (18)
C9—N1—C10—C11	−172.93 (12)	C16—C15—C17—C18	−171.68 (16)
C13—N1—C10—C11	3.78 (17)		

### Hydrogen-bond geometry (Å, °)

**Table:**

*D*—H···*A*	*D*—H	H···*A*	*D*···*A*	*D*—H···*A*
N2—H1N2···O1^i^	0.81 (2)	2.09 (2)	2.8789 (16)	163 (2)

Symmetry codes: (i) −*x*+3/2, *y*−1/2, −*z*+1.
